# GWAS Study Applied to Phenotypically Slow Growth Strains of *Listeria monocytogenes* Workflow and Outcome

**DOI:** 10.3390/microorganisms13092011

**Published:** 2025-08-28

**Authors:** Maria Elisabetta De Angelis, Giovanna Alessia Robbe, Cesare Cammà, Massimo Ancora, Serena Bosica, Mattia Ferrara, Marina Torresi, Frank M. Aarestrup, Alexander Gmeiner, Narong Nuanmuang, Patrick Murigu Kamau Njage, Chiara Bravaccini, Viviana Belardo, Chiara Di Iorio, Silvia Di Zacomo, Paolo Fazii, Francesco Pomilio, Pimlapas Leekitcharoenphon

**Affiliations:** 1 Istituto Zooprofilattico Sperimentale dell’Abruzzo e del Molise “G. Caporale”, 64100 Teramo, Italy; de.angelis88@outlook.com (M.E.D.A.); c.camma@izs.it (C.C.); m.ancora@izs.it (M.A.); s.bosica@izs.it (S.B.); m.ferrara@izs.it (M.F.); m.torresi@izs.it (M.T.); f.pomilio@izs.it (F.P.); 2 Department of Bioscience and Technology for Food, Agriculture and Environment, University of Teramo, Via Balzarini 1, 64100 Teramo, Italy; 3 Research Group for Genomic Epidemiology, National Food Institute, Technical University of Denmark, 2800 Kgs. Lyngby, Denmark; fmaa@food.dtu.dk (F.M.A.); algm@food.dtu.dk (A.G.); narong.nu@up.ac.th (N.N.); panj@food.dtu.dk (P.M.K.N.); pile@food.dtu.dk (P.L.); 4 UOC Food Microbiology Unit, Istituto Zooprofilattico Sperimentale del Lazio e della Toscana “M. Aleandri”, 00178 Rome, Italy; chiara.bravaccini@izslt.it (C.B.); viviana.belardo@izslt.it (V.B.); 5 UOC “Microbiologia e Virologia Clinica”, Presidio Ospedaliero “Spirito Santo”, Via Fonte Romana 8, 65123 Pescara, Italy; chiara.diiorio@asl.pe.it (C.D.I.); silvia.dizacomo@asl.pe.it (S.D.Z.); paolo.fazii@asl.pe.it (P.F.)

**Keywords:** *Listeria monocytogenes*, food, clonal complex, enrichment

## Abstract

*Listeria monocytogenes* (*Lm*) is a serious public health foodborne pathogen cause of listeriosis, usually in elderly, pregnant and immunocompromised people, linked to consumption of contaminated food, especially ready-to-eat (RTE) products. Different protocols can be used to detect *Lm*, and ISO11290-1:2017 is the reference method in Europe. Through molecular techniques such as whole genome sequencing (WGS) it is possible to discriminate between *Lm* strains, which are unequally distributed between clinical cases, food or food related environments, probably also due to enrichment step bias towards some *Lm* serogroup (IIa) compared to IVb. In the present work a set of *Lm* strains, detected in clinical cases and food, was investigated to define *Lm* strains growth ability after incubation in Half Fraser broth, and Genome Wide Association Studies (GWAS) applied to correlate the growth phenotype traits to presence of relevant genes. GWAS enabled the identification of a more relevant cassette of genes associated to a holin region of bacteriophage A118 and the determination of the distribution of relevant genes, highlighted from GWAS analysis within a population of *Lm* IVb and IIa.

## 1. Introduction

Listeriosis, caused by the etiological agent *Listeria monocytogenes* (*Lm*), is an important zoonotic disease. *Listeria monocytogenes* is a Gram-positive, facultative intracellular pathogen and the causative agent of listeriosis, a severe foodborne infection with high hospitalization and mortality rates, particularly among immunocompromised individuals, the elderly, pregnant women and neonates. The disease causes gastroenteritis, abortion and neurological syndromes and can lead to death in people with an compromised immune system [1]. It is a ubiquitous and heterogenous species, and many studies tried to find a correlation between *Lm* strains and their distribution in the environment. Indeed, *Lm* can be present in farms, the food industry and in a wide range of animals, including humans, who can become infected by ingestion of contaminated food. Generally, *Lm* is reported in different food sources, but the most relevant one is Ready-to-eat (RTE) foods.

In recent years, several listeriosis outbreaks have occurred in Europe, underscoring the pathogen’s public health significance. For instance, a large outbreak in Denmark between March and April 2024 was linked to contaminated RTE fish products, resulting in over 20 confirmed cases and five deaths. Similarly, a multi-country outbreak in 2022 was associated with contaminated smoked fish products, affecting multiple European countries. These events highlight the urgent need for improved surveillance, risk assessment and control measures for *L*. *monocytogenes* in the food chain [2].

Control measures for *Lm* are important in food industry in order to reduce the risk of transmission of listeriosis. In particular, cleaning and disinfection (C&D) standard operating procedures should be in place, as well as regular testing of food products and the environment, in particular aiming to establish effectiveness of sanitation procedures of the food industry working environment. Serogroup IVb of *Lm* is predominantly associated with clinical cases, while IIa is more frequently detected in food and food industry-related environment. Also, the distribution of clonal complexes (CCs) is uneven: for instance, CC1, CC2, CC4 or CC6 are often isolated in clinical cases rather than CC9 and CC121, usually detected in the food and food industry [3]. Detecting different strains of *Lm* is more challenging when multiple strains contaminate one sample simultaneously.

Different protocols are available to detect *Lm* in food and environmental samples, but in the European Union ISO 11290-1:2017-1 [4] is the reference method for *Lm* and *Listeria* spp. detection, and ISO 11290-2:2017-2 [5] is the reference method for enumeration. In agreement with that, the two-step enrichment broths used for the detection of *Lm* strains are the Half Fraser broth for the first enrichment and after 25 ± 2 h the Fraser broth in the second enrichment.

Given this, a potential bias was investigated towards strains of *Lm* usually adapted to food and the environment, rather than strains more likely to be involved in clinical cases, hence less represented in food and in the food producing environmental samples. The evaluation of growth ability of different serogroups of *Lm* strains in broths used for the enrichment step revealed that some strains were able to outgrow others when together in a mixed culture [6].

Furthermore, strain heterogeneity of *Lm* can influence its detection in food, clinical and environmental samples [7]. Additionally, other factors influencing *Lm* detection can be the stress state of the cells, for examples viable but not culturable cells (VBNC) [8].

After all, detection of *Lm* can be challenging, especially in heavily contaminated food, but pure culture of the strain is essential in case of listeriosis outbreaks to track the source of the infection and prevent the spread of the disease. This is more complicated considering the possibility of a sample contaminated with a mixture population of *Lm* belonging to different serogroups and different CCs. The complexity and heterogeneity of this pathogen at the genomic level is indeed an important challenge. The Whole Genome Sequencing (WGS) analytical method allows the collection of a wide number of data correlated to genomic sequences. It can be used for different aims, such as a deep discrimination of strains and clusters within the same group and determining if a strain belongs to the cluster cause of an outbreak or not [9]. Also, analysis of genomic sequences associated to specific phenotypes through genome wide association study (GWAS) can aid to investigate the genes involved in observation of a specific phenotype trait. Widely used, this investigation method was used previously in an Italian outbreak in the Marche region in 2015 to demonstrate the association between clones causing the disease and those causing the re-infection [10].

The aim of the present work was to study a set of *Lm* strains, mostly detected in clinical cases and one strain from food, to define growth ability after 24 and 26 h of incubation at 30 °C. Also, through further analysis, we investigated a workflow in order to correlate the growth phenotype traits of tested strains to the presence of relevant genes through GWAS and determine the distribution of relevant genes, highlighted from GWAS analysis within a population of *Lm* IVb and IIa.

## 2. Materials and Methods

### 2.1. Data Collection and Preparation

A total of 251 sequences of *Lm* [Table S1 in Supplementary Materials] were used in the present study.

In detail, strains were selected between those available from clinical cases and associated food for a total of 41 isolates (one food and 40 clinical), collected between 2015 and 2020, were tested at Istituto Zooprofilattico Sperimentale dell’Abruzzo e del Molise (IZSAM) in order to assess the growth potential of *Lm* after 24 and 26 h of incubation. Their sequences, together with other 127 sequences from strains linked to food producing environments, previously reported in Guidi et al. [11], were downloaded from National Reference Centre for whole genome sequencing of microbial pathogens: database and bioinformatic analysis (GENPAT), for a total of 168 sequences.

A total of 83 sequences were downloaded from the National Center for Biotechnology Information (NCBI) selected from bibliography using the parameters: growth of *Lm* in half Fraser broth or Brain heart infusion broth (IZSAM) and stress response to cold storage or treatment (between +7 °C and −20 °C).

For details of sequences used in the present study refer to Table S1 in Supplementary Data.

### 2.2. Growth Potential Evaluation

#### 2.2.1. Stocks Preparation

As above reported, 41 strains were tested for growth potential. In agreement with ISO 11290-2:2017-2 enumeration of Lm strains growth was done at 24 and 26 h. Strains stored at −80 °C in cryovials were streaked on ALOA agar plate (Liofilchem^®^, Roseto degli Abruzzi, Italy) and incubated at 37 °C for 24 h. After incubation, a single colony was streaked on Blood Agar (Liofilchem^®^, Italy) and incubated at 37 °C for 24 h to check purity of the microbank, and after incubation an isolated colony was used to contaminate 10 mL of Brain Heart Infusion Broth (BHI) (Liofilchem^®^, Italy) and incubated at 37 °C for 24 h. After 24 h, 100 μL of the contaminated broth were inoculated in 10 mL of BHI broth and incubated at 30 °C for 24 h. After 24 h of incubation, an optical density (OD600) measurement of the solution was performed and broth with OD600 > 0.125 was serially diluted four times and enumerated, and at the same time 1 mL stocks of the broth was dispensed in 1.5 mL or 2 mL Eppendorf tubes and then frozen at −20 °C to be used at a later time.

#### 2.2.2. Inoculum Preparation and Contamination of Half Fraser Broth

A known concentration stock was used to obtain a final concentration of about 10^3^ cfu/mL, which was used to contaminate the Half Fraser broth (1:10 ratio) (IZSAM), in agreement with ISO 11290-1:2017-1. Hence, 1 mL of the prepared inoculum was used to contaminate 9 mL of Half Fraser broth supplemented with ferric ammonium citrate as suggested by the producer, for a final expected contamination level of about 100 CFU/mL.

#### 2.2.3. Enumeration and Growth Rate

Contaminated broth was enumerated at time of preparation, and, according to ISO 11290-2:2017-2 maximum and minimum incubation time, after 24 h and 26 h at 30 °C. Enumeration was determined using the excel software MICROINCERT MAXI rev. 6 (https://sites.google.com/site/incertezzamicro/informatica-per-il-laboratorio-2/microincert-maxi, accessed on 18 December 2023). The experiment was conducted in duplicate.

#### 2.2.4. Graphics Analysis

R studio software (version 4.4.1) was used to elaborate the data graphics.

In particular, data analysis was performed using library(readxl), library(dplyr), library(tidyverse), library(writexl), library(tidyr), library(data.table); graphics using library(ggplot2); heatmap visualization using library(pheatmap).

Finally, ITOL gene presence/absence representation was done using table2itol.R—Rscript script for generating input files for iTOL.

### 2.3. DNA Extraction, Sequencing and Bioinformatic Analysis

DNA was extracted using a Maxwell^®^ 16 tissue DNA purification kit (Promega Italia Srl, Milan, Italy) according to the manufacturer’s protocol for strains of *Lm* collected before 2018, and the DNA purity was checked by NanoDrop2000 (ThermoFisher Scientific, Waltham, MA, USA). DNA extraction was performed using QIAamp^®^ DNA Mini Kit (Qiagen, Hilden, Germany) following the manufacturer’s protocol with minor modifications according to a previous study [12]. DNA quantity and quality were evaluated with a Qubit fluorometer (Thermo Fisher Scientific, Waltham, MA, USA) and Eppendorf BioSpectrometer fluorescence (Eppendorf s.r.l., Milano, Italy) for strains isolated after 2018. Starting from 100–500 ng of input DNA, the Illumina DNA Prep kit (Illumina, San Diego, CA, USA) was used for library preparation according to the manufacturer’s protocols. WGS was performed on the NextSeq^®^ 500 platform (Illumina^®^, San Diego, CA, USA) with the NextSeq 500/550 mid-output reagent cartridge v2 (300 cycles, standard 150 bp paired-end reads). For the WGS data analysis, an in-house pipeline [13] was used. The trimming step of raw reads was performed using Trimmomatic [14] and a quality control check of the reads using FastQC v.0.11.5 (https://github.com/s-andrews/FastQC). De novo assembly of paired-end reads was carried out using SPAdes v3.11 (https://github.com/ablab/spades) [15] with default parameters for the Illumina platform 2 × 150 chemistry. QUAST v.4.3 (https://github.com/ablab/quast) was used for checking the quality of the genome assemblies. Assembled genomes of the strains selected from reference public databases were downloaded from NCBI and assembled valued with QUAST v.4.3. All the genomes that met the quality parameters recommended by Timme et al. [16] were used for the subsequent analysis steps. Multilocus sequence typing (MLST), based on the Pasteur scheme, was used to characterize *Lm* strains and detect the sequence type (ST) and clonal complex (CC) querying the Pasteur Institute platform (https://bigsdb.pasteur.fr/listeria/ (accessed on 10 October 2022). [Project number of used sequences is reported in Table S1 in Supplementary Materials].

Core genome MLST (cgMLST) of *Lm* was calculated according to the Institut Pasteur’s scheme of 1748 target loci using the chewBBACA allele-calling algorithm [17]. Genomes with at least 1660 called loci (95% of the full scheme) were included in the analysis.

### 2.4. Genome Annotation and GWAS

Gene annotation was carried out using Prokka [18], with default parameters. GFF files produced by Prokka, including sequences and annotations, were used to determine the pangenome with Panaroo [19]. The genes were considered present or absent based on Panaroo prediction using a 98% identity cut-off after annotation correction and CD realignment.

GWAS analysis was performed using Scoary [20]. A trait presence absence file was created for Scoary analysis to determine more relevant genes involved in the phenotype observation. To reduce the genomic heterogeneity between strains of *Lm* and increase detection of association between genetic and phenotypic traits, despite the low number of tested strains, analysis was focused on *Lm* CC1 sequences. A score of 0 was applied, and the strains defined as *slow* if the strains did not reach an average growth of 8 CFU_log10_/mL after 24 h incubation, while a score of 1 was applied to strains defined as *normal* if strain growth was over 8 CFU_log10_/mL after 24 h incubation. Not applicable (NA) was used for strains selected from NCBI, for which growth value were unknown.

### 2.5. Phylogenetic Analysis

Phylogenetic and gene presence/absence representation was done using iTOL [21]. SNP analysis was performed through an IZSAM in-house pipeline using SNIPPY [22], and a variant call format (.vcf) file was generated for each strain, then the multiple .vcf files were merged into a single .vcf, using BCFTOOLS-merge command [23], and representation in newick format tree was elaborated after SNP analysis using CSI phylogeny [24].

### 2.6. Gene Alignment and Protein Similarity Visualization

Clinker was used to visualize genes evaluated as most relevant to phenotype observation by Scoary [25]. Protein similarity and gene similarity between strains was performed using tBLASTn and BLASTn, respectively. Heatmap was elaborated using R Studio software.

### 2.7. Protein Translation and Function

Genes scored as more relevant from GWAS analysis were investigated for protein translation and function using the UniProt BLAST online tool [26].

## 3. Results

**Figure 1:**
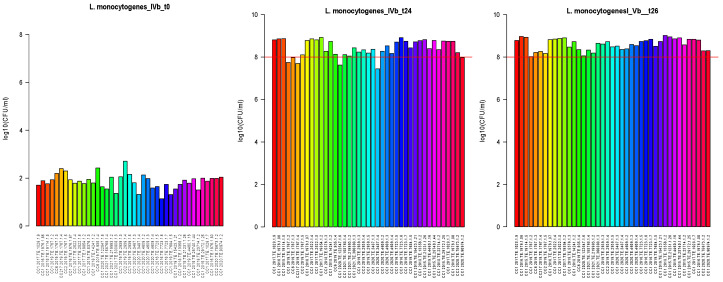
Results of *Lm* growth rate after inoculation of half Fraser broth (T0, first bar plot) and after 24 and 26 h of incubation, respectively t24 (second bar plot) and t26 (third bar plot). All strains tested (n = 41) are reported on the x-axis, and the y-axis shows the concentration of *Lm* at different times. Lines in red delimit a growth threshold of 8.0 CFU_log10_/mL.

The growth ability of 41 strains was measured after 24 and 26 h, and the results were evaluated. The inoculum preparation at contamination was about 1.87 CFU_log10_/mL, with a maximum of 2.71 CFU_log10_/mL and a minimum of 1.14 CFU_log10_/mL. After 24 h of incubation at 30 °C, the median value was about 8.20 CFU_log10_/mL with a maximum of 8.74 CFU_log10_/mL and a minimum of 7.60 CFU_log10_/mL. Among the strains tested, four CC1, one strain CC217 and one strain CC6 did not reach 8.0 CFU_log10_/mL after 24 h incubation, but all the strains reached 8.0 CFU_log10_/mL after 26 h incubation, Figure 1.

**Figure 2:**
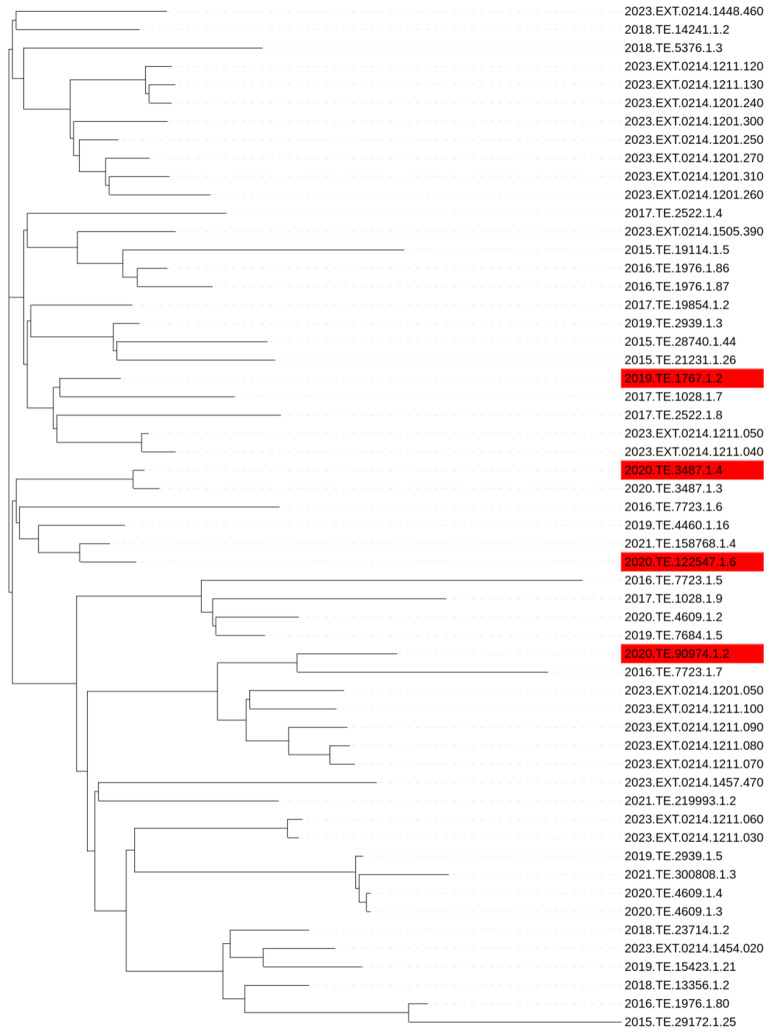
SNP based phylogenetic tree of *Lm* CC1 used in the present study. Red labelled strains are phenotypically slow growth.

CC1 strains that did not reach 8.0 CFU_log10_/mL after 24 h of incubation were considered as the slow growth phenotype. GWAS analysis was performed on all the tested CC1 strains (n = 35) and a set of CC1 strains selected from NCBI (n = 21). No phylogenetic clustering was observed between slow and normal growth strains after performing SNP analysis Figure 2.

**Figure 3:**
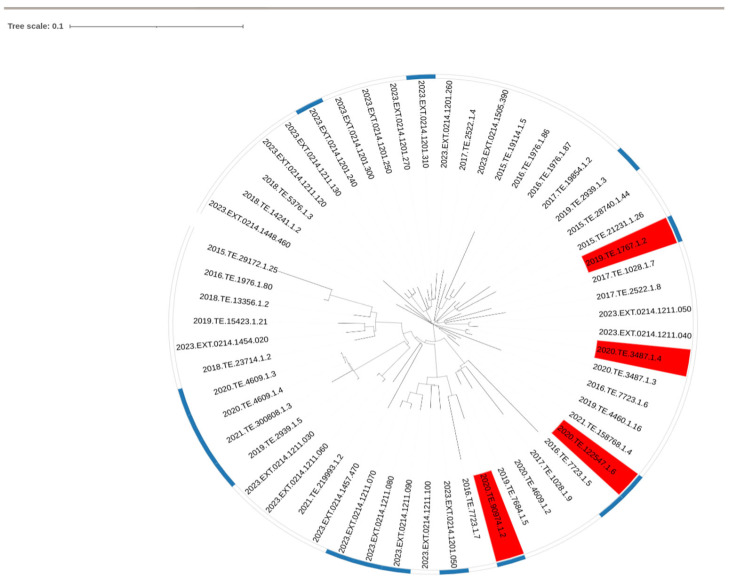
Visualization of strains showing presence of relevant gene highlighted by Scoary *group_2881* (in light blue strip). In red were highlighted strains showing phenotypically slow growth compared to other strains consider to be normal in growth.

**Figure 4:**
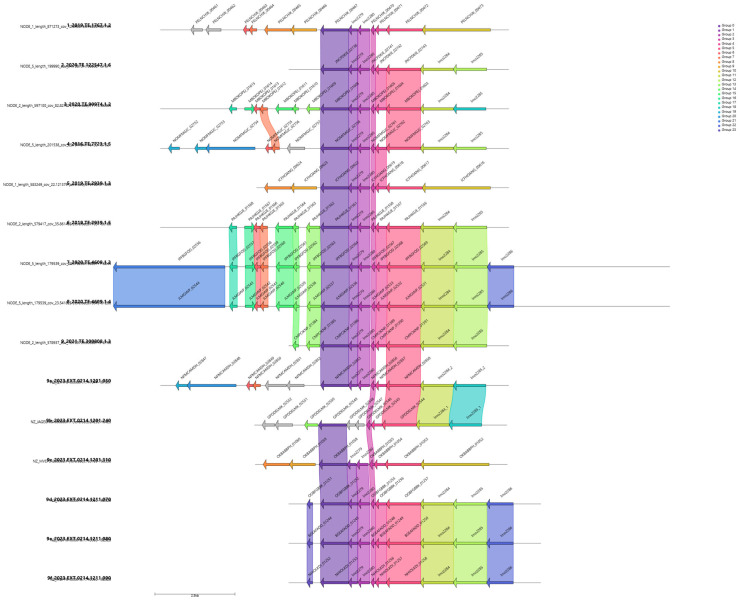
Gene alignment visualization for the gene *group_2881*, highlighted as relevant in Scoary analysis and gene similarity. Top four strains are those reported as slow growth.

Genome annotation and analysis found a total of 3844 genes and in 3 out of 4 slow growth strains, one relevant group of genes, namely “*group_2881*”, was highlighted by the GWAS analysis, Figure 3. *Group_2881* was present in 15 strains out of 56 (37% of the strains). A gene cassette composed of a total of six genes, namely *group_2881*, *lmo2279*, *lmo2280*, *lmo2783*, *group_3146* and *lmo2283,* was identified through gene visualization of nearby genes of the group highlighted by Scoary, Figure 4.

**Figure 5:**
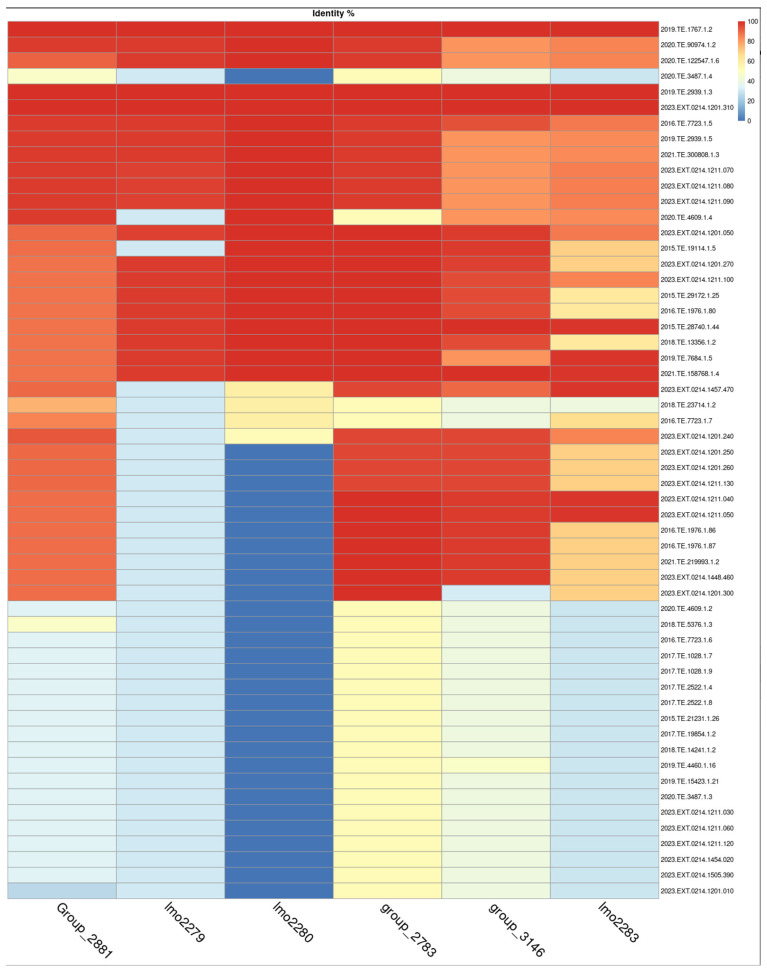
Heatmap of tBLASTn gene similarity. Top four strains are those reported as slow growth.

The cassette of genes was present in other strains with normal growth, therefore tBLASTn was used to obtain the percentage identity among genes starting from translated proteins, and heatmap was used to visualize the results of the identity between selected genes Figure 5.

A pattern in genes *lmo2279* and *lmo2280* was evident and it was possible to highlight a decreasing pattern between genes belonging to slow growth strains compared to normal growth strains. Considering a similarity identity under 50% as indicative of gene absence. A similarity greater than 90% in gene *lmo2280* gene was observed in 23 out of 56 strains (41%), while it was absent in 29 strains out of 56 (51%). In contrast, *lmo2279* was present in 21 strains out of 56 (37%) with a similarity identity over 90% and present with a similarity identity under 50% in 35 strains out of 56 (62%).

**Figure 6:**
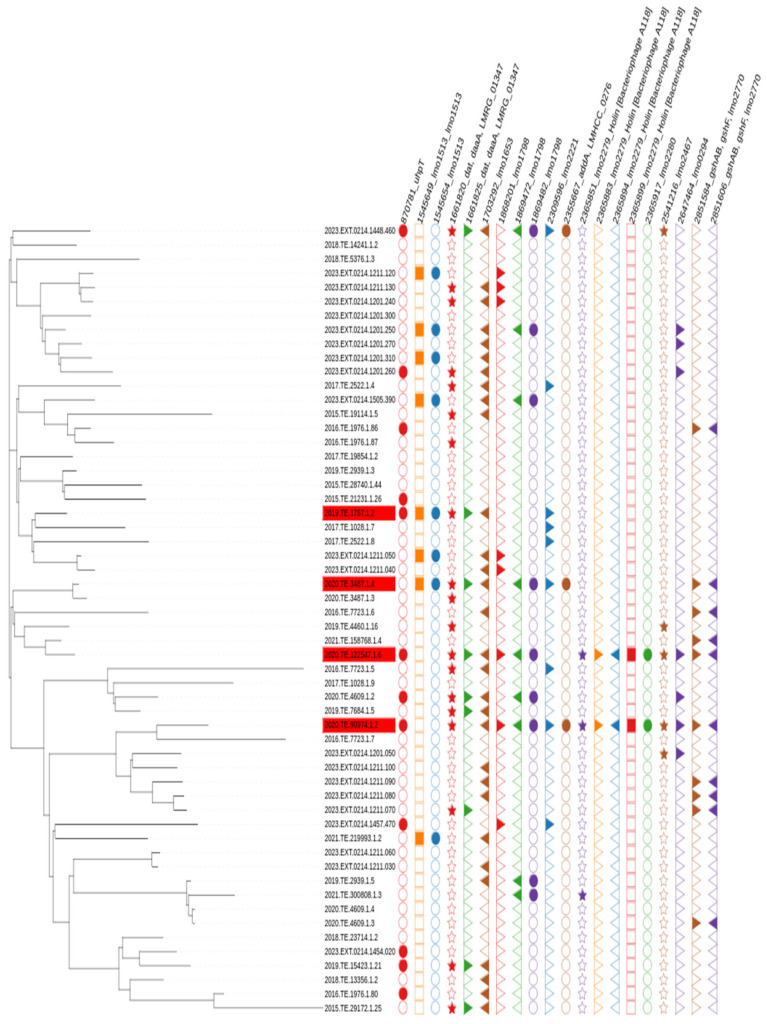
SNP analysis visualization of relevant mutation between strains considered slow growth (in red) compared to other strains.

GWAS at SNP level allowed the identification of a total of 20 relevant mutations in genes *uhp_T*, *lmo1512_LMRG_01347*, *lmo1653*, *lmo1798*, *lmo2221_LMHCC_0276*, *lmo2467*, *lmo0294* and *lmo2270*, and, noticeably, mutations in *lmo2280* and *lmo2279* genes were present in only two of the phenotypically slow growth *Lm* CC1, but not in other strains, Figure 6.

**Figure 7:**
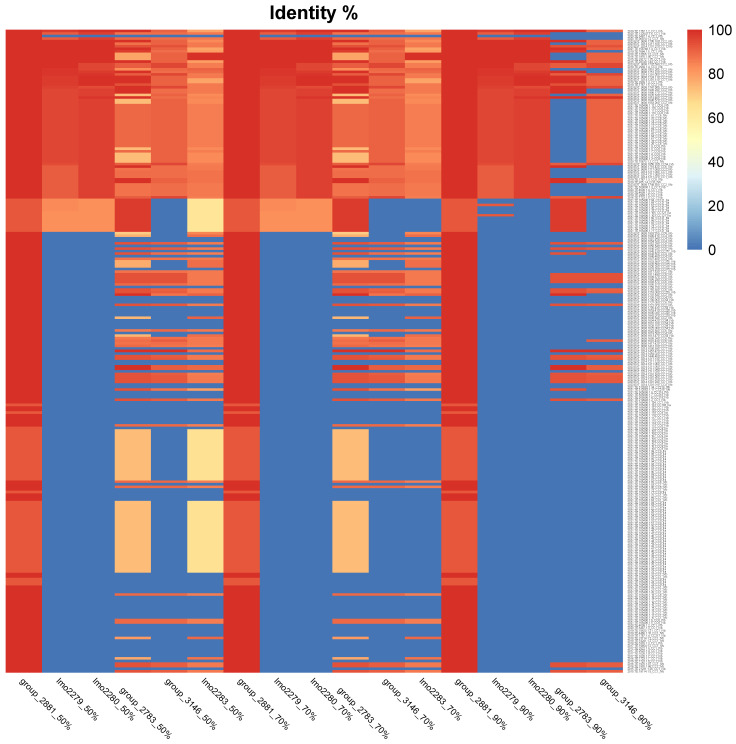
Heatmap of tBLASTn gene similarity of 251 strains. Top four strains are those reported as slow growth.

Finally, to evaluate the distribution in different strains of *Lm* of the cassette genes highlighted as more relevant thorough GWAS analysis (*group_2881*, *lmo2279*, *lmo2280*, *lmo2783*, *group_3146* and *lmo2283*), more strains of *Lm* IVb and IIa were analyzed, and BLASTn at different percentages of identity (50%, 70% and 90%) was performed and visualized through a heatmap, Figure 7.

Among the highlighted genes, *group_2881* was the most shared gene within the 251 analyzed strains, with a similarity over 90%, followed by gene *group_2783*. *Lmo2279* and *lmo2280* were absent in around the 70% of the analyzed strains, followed by gene *group_3146* and the *lmo2283* gene and, last, *group_2783*. The use of different thresholds for the analysis (50–70% and 90%) had no evident impact in case of *group_2881*, *lmo2279* and *lmo_2280*, compared to the rest of the investigated genes.

Among the 251 strains analyzed, 68 strains belong to serogroup IIa, and 183 to serogroup IVb. *lmo2279* was present in 13 strains IIa, with a similarity between 50 and 90% and only IVb strains with a similarity over 90%, at a 70% threshold, while *lmo2279* was present in 2 strains IIa and 65 IVb with over 90% similarity, at 90% threshold, *lmo2280* instead was absent in all IIa, while it was present only in 65 strains IVb (Table 1).

BLAST of highlighted genes from GWAS analysis, *lmo2280* and *lmo2279* matched as holins bacteriophage A118 proteins associated to a canonical holin pathway for lytic cycle [27].

## 4. Discussion

*Lm* is an important foodborne pathogen responsible of listeriosis. Its detection in contaminated food can be difficult, especially in complex food contaminated by multiple bacteria species. Furthermore, detection rate in food or the environment associated to the food industry of clonal complex (CC) usually associated to clinical cases, (serogroup IVb), like CC1, has been reported as underrepresented when compared to other CCs normally detected in food like CC121 and CC9 (serogroup IIa) [3]. In our study we investigated growth ability of *Lm* strains at 24 h and 26 h in a set of clinical cases and one strain isolated in food, and we applied the use of Genome Wide Association (GWAS) to investigate the presence of genes that may explain normal growth traits compared to strains showing a slow growth phenotype.

Among tested strains of the same serogroup of *Lm* (*IVb*), it was possible to notice a slower growth ability in four strains, which did not reach the average growth of 8 CFU_log10_/mL within 24 h compared to other strains. This is supported by other studies, reporting differences in growth ability of strains belonging to same serogroup [6]. Moreover, a recent study highlighted differences in replication capacity among strains within the same serogroup and demonstrated that external factors, such as the cellular stress and physiological state can influence strain recovery and detection [8]. This being said, a limitation of the study was determined by the low number of isolates tested. Unfortunately, a larger set of clinical isolates were not available for deeper investigation. It would be recommended to collect more strains to improve reliability of the results.

Genome investigation through GWAS analysis can be an important tool to associate loci in the genome to a phenotype [18] and aid to unveil genes behind specific phenotypes. Therefore, further investigation was conducted to associate phenotypically slow strain traits to relevant genes. To reduce the genomic heterogeneity between strains of *Lm* belonging to different serogroups and to make easier the detection of the association between genetic variants and phenotypic traits, the analysis was focused on strains of *Lm* CC1, which is one of the CCs that is less represented in clinical cases compared to food detection. Due to the limited number of tested strains, additional CC1 strains were selected from the literature, but a challenge of the present study was the difficulty of retrieving similar experiments. Therefore, we based the approach on the assumption of a normal growth rate for strains selected from the literature for which experiment settings were not comparable to ours, and, in the Scoary input file, growth values were marked as ‘Not Applicable’ due to the lack of experimental data. It could be interesting to confirm this hypothesis by testing more strains and including those used for the analysis as well.

It is important to emphasize that additional tests and a larger set of strains are required, as different outcomes of the analysis may emerge from further experimental results. In fact, GWAS is a powerful tool for this purpose; however, the number of strains to examine in order to have statistically significant results is a parameter to take into consideration when interpreting the results [28].

Pangenome analysis detected 3488 genes; however, the study highlighted only one gene as significantly more relevant. This gene is itself part of a group of genes that might explain the phenotype. The group of genes, reported in slow growth strains, were shared with other normal growth strains. Moreover, those genes were absent in one of the four slow growth strains. Further investigation was performed on nearby genes from the highlighted one by Scoary to evaluate differences within this group of genes between slow and normal growth strains. A cassette of genes was found to be shared between slow and normal growth strains and the pattern of gene similarity in the cassette showed differences between them.

Within the cassette of genes highlighted in the GWAS analysis and associated to an holin region of bacteriophage A118, two genes were noticeably reported as more relevant, namely, the *lmo2280* and *lmo2279* genes. Furthermore, mutations were also highlighted by the SNP analysis in slow growth strains compared to normal growth strains, suggesting the relevance of the importance of these genes in association to the slow growth phenotype and the need for further investigation. Bacteriophage A118 is a temperate phage isolated in *Lm*, and holin proteins form unspecific lesions within the host cytoplasmatic membrane [29] and mediate the lysis of the bacteria [30]. Further analysis and more tests, especially with a larger set of sequences, is necessary to confirm and to properly evaluate the involvement of this phage and its role in *Lm* growth. Unfortunately, the low number of sequences used for the study makes impossible to statistically validate the outcome of the study; therefore, more tests are needed for confirmation.

It is well established that genes associated with *Lm* phages can play a role in enhancing disinfectant resistance [31] or inhibiting *Lm* growth, Consequently, their application in the food industry as alternative methods of disinfection is under evaluation [32]. Hence, even if not statistically significant at the moment, more tests are advised to confirm these findings.

Finally, tBLASTn was fundamental in calculating the identity percentage of the genes between slow and normal growth strains and the comparison of the cassette between strains with different growth ability, which highlighted a reduced gene similarity between the examined strains, as in Figure 4. The same pattern was consistent even when it was analyzed in comparison with strains of *Lm* IVb, other than CC1 from different sources. BLASTn highlighted absence of gene *lmo2280* and *lmo2279* in most of the strains collected from bibliography (about 70%). Hence, despite the presence of these genes, not only in slow growth strains but in normal growth strains as well, a relevant difference can be noted within the distribution of these two genes between strains of *Lm*. Strains used for the experiments were collected from clinical cases, while the genomic sequences used for bioinformatic analysis were collected within the food industry environment, in particular poultry [11]. A better representation of the different sources should be considered using a wider range of strains and additional growth assays should be performed should be performed, considering also the role played by phage, their gene distribution among strains of *Lm* and how they can influence the microorganism phenotype [32].

It is important to highlight the limitations of the present study. First, the limited number of tested strains are not large enough to statistically support our founding. Second, increasing the number of the sequences tested can change the outcome of the GWAS results, given that the collection of genomic sequences from a publicly available repository can increase the number of sequences analyzed. However, the experimental settings, at least in our case, were not comparable; therefore, much information was unknown, and analysis was based on general assumption. From this perspective, more strains should be tested to increase confidence in the results and correctly make inferences.

## 5. Conclusions

In conclusion, despite the small number of sequences tested two main conclusions can be drawn. First, there is notable variability in growth capacity among *Lm* strains belonging to the same serogroup. Second, GWAS analysis was able to highlight differences in a gene cassette (*group_2881*, *lmo2279*, *lmo2280*, *lmo2783*, *group_3146* and *lmo2283*), although no unique gene was found. In addition, tBLASTn results highlighted absence of *lmo2280* and *lmo2279* between strains from CC1 and other CCs, and SNP analysis confirmed mutations in these genes in two of the four strains phenotypically associated with slow growth.

Noteworthily, relevant genes *lmo2280* and *lmo2279*, from GWAS analysis, were associated with the phage insertion region, which could play a role within *Lm* phenotype expression. However, further studies are necessary to confirm this hypothesis and clarify the functional impact of these phage-associated genes on bacterial physiology. In conclusion, this work highlights a different approach for genomic analysis in datasets with limited sample sizes. Despite the limited number of sequences tested, more information and genome strains can be used for analysis when taken from the literature. Moreover, our results reinforce the utility of GWAS as a powerful tool for exploring genotype–phenotype associations, even under constrained experimental conditions. Nevertheless, these findings must be interpreted with caution and validated through additional experimental work. In our case, further study should be done to understand gene distribution within a *Lm* population of which growth ability is known, and confirm if phage proteins could influence, positively or negatively, the microorganism growth.

## Figures and Tables

**Table 1:** Percentage of genes present out of 251 strains analyzed based on similarity percentage and thresholds of 50%, 70% and 90% used for analysis in tBLASTn.

Gene	Threshold (%)	n. Absent	Absent %	n. Similarity 50–90%	Similarity 50–90 %	n. Similarity > 90%	Similarity > 90%
Group_2881	50.00%	0	NA	0	NA	251	100%
Group_2881	70.00%	0	NA	0	NA	251	100%
Group_2881	90.00%	0	NA	0	NA	251	100%
lmo2279	50.00%	173	69%	13	5%	65	26%
lmo2279	70.00%	173	69%	13	5%	65	26%
lmo2279	90.00%	184	73%	0	0	67	27%
lmo2280	50.00%	173	69%	13	5%	65	26%
lmo2280	70.00%	173	69%	13	5%	65	26%
lmo2280	90.00%	186	74%	0	0	65	26%
group_2783	50.00%	77	31%	113	45%	61	24%
group_2783	70.00%	77	31%	113	45%	61	24%
group_2783	90.00%	190	76%	0	0	61	24%
group_3146	50.00%	147	59%	34	14%	70	28%
group_3146	70.00%	147	59%	34	14%	70	28%
group_3146	90.00%	180	72%	0	0	71	28%
lmo2283	50.00%	77	31%	168	67%	6	2%
lmo2283	70.00%	138	55%	107	43%	6	2%
lmo2283	90.00%	240	96%	0	0	11	4%

## Data Availability

The sequencing data that support the findings will be available in National Center for Biotechnology Information (NCBI) BioProject under accession PRJNA1310828 following an embargo from the date of publication to allow for commercialization of research findings.

## Supplementary Materials

The following supporting information can be downloaded at: https://www.mdpi.com/article/10.3390/microorganisms13092011/s1, Table S1: CSV_file strains.

## Abbreviations

The following abbreviations are used in this manuscript:

*Lm*	*Listeria monocytogenes*
RTE	Ready-to-eat food
WGS	Whole Genome Sequencing
GWAS	Genome Wide Association Studies
C&D	Cleaning and disinfection
CCs	Clonal complexes
GENPAT	National Reference Centre for whole genome sequencing of microbial pathogens: database and bioinformatic analysis
NCBI	National Center for Biotechnology Information
MLST	Multilocus sequence typing
ST	Sequence type
cgMLST	Core genome MLST
NA	Not applicable
